# A novel technique to overcome fluid flow influence in carbon quantum dots/paper-based analytical devices

**DOI:** 10.1038/s41598-022-22837-2

**Published:** 2022-10-25

**Authors:** Sepideh Zoghi, Moones Rahmandoust

**Affiliations:** Protein Research Center, Shahid Beheshti University, Tehran, Iran

**Keywords:** Optical materials and structures, Natural hazards, Design, synthesis and processing

## Abstract

Paper-based analytical devices are promising choices for rapid tests and lab-on-chip detection techniques. Carbon quantum dots (CQDs), on the other hand, are biocompatible nanomaterials, which are industrially promising, due to their fast and cost-effective gram-scale synthesis techniques, as well as their significantly high and stable photoluminescence (PL) properties, which are durable and reliable over a year. However, there have been limitations in the entrapment of CQDs on cellulose papers in a way that their PL is not influenced by the flowing of the CQDs with the stream of analyte fluid, making the sensors less accurate at very low concentrations of liquid analytes. Therefore, in this investigation, a polyvinyl alcohol/alkaline-based method was systematically generated and developed to entrap CQDs inside a 3D crystalline matrix on paper, in a way that they can be used directly as probes for a simple drop-and-detect method. As a proof of concept, N/P-doped CQD on cellulose paper was used to make fluorescent paper-based analytical devices for identifying traces of Hg^2+^ of around 100 ppb. The designed sensor was tested over several months, to study its durability and functionality over long periods, for potential industrial applications.

## Introduction

Carbon quantum dots (CQDs) are a class of nanomaterials, famous for their significant photoluminescence (PL) properties, their fast and gram-scale synthesis techniques, as well as biocompatibility and ease of surface functionalization. They can emit a variety of different wavelengths, depending on their size, as the major influential factor, edge configuration, chemical structure, and heteroatom dopings, which are generally durable over several months^1^. This feature makes CQDs promising probes for an easy mix-and-detect analytical monitoring of various environmentally and biologically important analytes.

Polymers, on the other hand, are materials that are used in everyday life for a wide range of applications. The worlds of polymers and CQDs meet each other in various ways; polymers can be either used as precursors in the synthesis process of CQDs, or they can be used as surface modification tools or as supporting matrices to hold CQDs in them and make fluorescent composites. In recent years, CQDs were increasingly conjugated in polymer matrices and polymeric gels for different applications^2–4^, ranging from biological applications, such as bioimaging and drug delivery or anticancer agents and wound healing patches, to nonbiological applications, such as detection, supercapacitors, electrocatalysis and light-emitting diodes^5–9^.

Among natural polymers, cellulosic filter papers are generally regarded as promising scaffolds for being utilized in making diverse sensing and separation devices based on CQDs, or other functional guest substances. They have proper porosity, biodegradability, flexibility, as well as a large, natural, white-coloured surface area, providing the possibility of being functionalized as cellulosic materials with engineered properties^10^. In addition, cellulose is a chemically inert polymer. Hence, the natural polymer can host the immobilization of various functional substances, expecting no interactions between the host and the guest, such as other polymers, nanoparticles, proteins, DNA and small molecules^11^. Therefore, it is expected that a large spectrum of stimuli-responsive smart sensing and monitoring platforms for various disease biomarkers and environmental contaminations is achieved based on fluorescence signal reading^12–14^.

Development of hydrophilic cellulose or nitrocellulose-based devices began less than a decade ago to meet the increasing need for portable, and point-of-care medical diagnostic systems. The paper-based analytical devices (PADs) are easy to use and non-expert user-friendly and they can have tunable analytical performances. These devices typically consist of some hydrophilic paper detection pools which hold CQDs. As an extra advantage, when CQDs are embedded inside PADs, immobilization of the CQDs into the structure of the natural polymer scaffold contributes to the enhancement of their PL by preventing aggregation-induced fluorescence quenching. However, detection sensitivity is still a crucial factor that is required to be improved. When CQD-PADs are made by drying CQD drops on paper, the sensor’s fluorescence signal is affected by dropping water on the fluorescent spot. This phenomenon is observed because the CQD on the paper move with the flow of the dripped blank sample, making a darker region. Hence, it is necessary to stop them from sliding with the flow of liquid samples to achieve more accurate results, at low concentrations.

Throughout the following years, some scholars tried to address the CQD-entrapment issue. Copur et al. and Li et al. and Jiang et al. are three of the examples^15–17^. Li et al. designed another CQD-PAD using a hybrid platform of cellulose paper and polydimethylsiloxane (PDMS), in which CQDs were loaded on the paper and then transferred to grooves array of PDMS plates. They tested the performance of their sensor with folic acid, with a limit of detection of 0.28 μM. They also showed that these types of devices are far more sensitive than the common solution-based systems, making them candidates for a bright foreseeable future for practical applications in biosensing and clinical diagnosis^16^. In another approach toward achieving an in-situ platform, a colour-change monitoring strategy was employed for the detection of the Cr(VI). For that, a thickened CQD solution was dripped over the paper and let dry. The targeted analyte was then dropped over the test paper, showing a change in colour of the emission from yellow to blue under the UV light of 365 nm with the increasing concentration of Cr (VI)^18^. In 2022, the N-doped CQDs were integrated into a nitrocellulose membrane to develop a paper-based nanoprobe for Hg^2+^ detection. For that, the filter membrane was first washed with ethanol solution and water to remove its impurities and then it was immersed in CQDs solution for 30 min. The prepared PAD was then titrated with different concentrations of Hg^2+^ solution^19^. In the study, the as-prepared N-doped CQD-PAD was shown to be capable of in situ detection of Hg^2+^ in environmental water, and food, which makes the paper-based device valuable. However, finding reliable solutions to address the issue is still a matter of concern.

Hence, in this paper, a polyvinyl alcohol/alkaline-based technique was systematically generated and developed to entrap N/P-doped CQDs (NPCQDs) as fluorescent spots on paper and make fluorescent PADs for identifying traces of Hg^2+^ ion contaminations in water. The purpose of this investigation is to control the NPCQDs from flowing with the stream of analyte fluid, as it is poured on the fluorescent spots, using a simple drop-and-detect method. Hence, proper steps were taken to reduce the phenomena, termed here as the fluid flow influence (FFI). Finally, the designed sensor was tested over a 7-month period to study its durability and functionality.

## Experimental methodology

### Materials and instruments

All chemicals were purchased from Sigma Aldrich and Merck Chemical Companies, Germany. The average particle size and zeta potential were confirmed by the dynamic light-scattering method (DLS), (HORIBA model SZ-100, Kyoto, Japan). All experiments were conducted in three replications, at room temperature, at the scattering angle of 90°, using a 632.8 nm HeNe laser light source, with a maximum intensity of 10 mW. Bright-field high-resolution transmission electron microscopy (TEM) was conducted to analyse the morphology, particle size, and agglomeration behaviour. The NPCQDs were highly diluted in distilled water. The suspension was then homogenized in an ultrasonic bath for 5 min. Immediately after the ultrasonic treatment, micro-droplets were put onto 3 mm lacey carbon TEM grids (TED PELLA INC.) and left to dry for at least 30 min. Afterwards, the grids containing the NPCQDs were analysed in a JEOL 2200FS TEM, Tokyo, Japan, operating at 200 kV.

X-ray Photoelectron Spectroscopy (XPS) was performed using PHI 5000 Versaprobe, Physical Electronics, Minnesota, USA and the atomic force microscopy (AFM) instrument employed was the Easyscan 2 Nanosurf, Liestal, Switzerland. Fourier transform infrared spectroscopy (FTIR) (Thermo-Nicolet NEXUS 470, Illinois, USA) was employed to analyse the chemical bonds of the synthesized NPCQDs. The X-ray diffraction (XRD) spectrum was recorded by a DMAX-2500 diffractometer (Rigaku, Japan), using a Cu *Kα*1 X-ray radiation source, in the range of 2*θ* between 1° and 80°, to provide information about the crystalline structure of the particles. Ultraviolet–visible spectroscopy was used to characterize the optical properties of the CQDs (PerkinElmer’s LAMBDA 950 UV/Vis/NIR Spectrophotometer, USA) in optical glass cuvettes, path length 10 mm. A photoluminescence spectrophotometer (PerkinElmer LS45, Massachusetts, USA) was used. In the study of the optical properties, the samples were diluted at different concentrations each, to an optical density of about 0.1, to minimize the re-absorbance effect^20^. For the purpose of capturing images from the samples prepared on cellulose paper, the INTAS Gel documentation hood imager (Göttingen, Germany) was used together with iPhone Pro Max 11 mobile camera to photograph the carbon dots emissions. The obtained images were analysed using the ImageJ Android application (IJ_Mobile^®^, version 1.1, 2013).

### Synthesis of NPCQDs

N/P-doped CQDs were prepared in accordance with previously described methods with minor modifications, using a single-step hydrothermal method^1^. Citric acid monohydrate (0.30 g) and ammonium phosphate dibasic (0.75 g) were well dissolved in 20 ml of distilled water and were heated hydrothermally in a Teflon-lined reactor at 180 °C for 4 h. The obtained dark brown solution containing the NPCQDs was neutralized to pH 7.0, after reaching room temperature. Then, it was centrifuged at 20 k rpm for 15 min and filtered to remove large particles.

### Device preparation

#### Study of selectivity

Initially, NPCQD solution was diluted four times using distilled water and 3-μl drops of the diluted solution were poured onto a Whatman cellulose filter paper grade 591. Then, 0.5 μl of seven various 2 mM metal cations, namely, Pb^2+^, Hg^2+^, Cu^2+^, Fe^3+^, Fe^2+^, Ca^2+^, Zn^2+^, were poured over the NPCQD fluorescent spots, to study the selectivity of the NPCQDs. The blue PL emission of the NPCQD was better quenched by the Hg^2+^ ion compared to other metal ions, as shown in the inset of Fig. S1-a, due to the formation of the mercury hydroxide complex on the surface of NPCQDs^21^.

#### PVA concentration adjustment

In the next step, PVA solutions of different concentrations, i.e. 0% (distilled water), 1%, 2%, 4%, and 10%, were prepared and then mixed with the NPCQDs solution at a 3:1 volume ratio, diluting the stock of the NPCQD sample four times. The mixture was then placed in a 30 °C sonication bath for 20 min, making the NPCQD-PVA (NP-PVA) composite solution. Then, 3 µl drops of the achieved NP-PVA nanocomposites were dropped on a new filter paper and let dry at ambient temperature for about 30 min, making the fluorescent spots on the paper.

A matrix of 3 rows and 5 columns was printed on the filter paper, in which, the three rows contained NP-PVA fluorescent spots, NP-PVA fluorescent spots plus 0.5 µl drop of water, and NP-PVA fluorescent spots plus 0.5 µl drop of Hg^2+^ (10 mM). The matrix columns on the filter paper contained NP-PVA fluorescent spots at five different PVA concentrations as introduced. The matrix was designed so, to analyse the effect of PVA density on the emission intensity of the NP-PVA nanocomposite and to study the effect of pouring water (FFI) and Hg^2+^ drops on the emission of the fluorescent spots.

#### Alkaline treatment

After choosing the best PVA density, alkaline treatment was conducted to control the FFI. For that, a paper containing a row of three 3 µl drops of the optimum NP-PVA fluorescent spots was soaked in NaOH 6 M for 5 min was then immersed in water for at least 10 min. Finally, the paper was placed flat in the oven and was dried at 60 °C for about 15 min. It should be noted that if the paper turns yellow after drying, it indicates that NaOH residue is still present on the paper. Afterwards, 0.5 µl drops of water and Hg^2+^ (10 mM) were poured on two of the alkaline-treated NP-PVA fluorescent spots, separately.

Finally, to study the effect of the number of NP-PVA layers on the emission intensity of the alkaline-treated fluorescent spots, the above step was repeated on another filter paper with 3 columns, each containing one, two, and three layers of the NP-PVA, before alkaline treatment. The fluorescent spots were finally stained by 0.5 µl drops of water and Hg^2+^ (10 mM).

### Device performance

At the final stage, the NP-PVA fluorescent PADs were exposed to various Hg^2+^ concentrations of 0.01, 0.05, 0.1, 0.5, 1, 2, 5, and 10 mM to study the quenching capability of the designed PAD. Furthermore, in order to study the performance of the device over longer durations, the NPCQD paper nanocomposites were created at various pHs and kept at cold room and room temperature, for over 6 months and their emissions were recorded timely starting from the first day, under 360 nm UV emission.

## Results and discussion

### Material characterization

According to the obtained UV–visible spectrum of the synthesized NPCQDs, under an excitation wavelength of 360 nm, NPCQDs emit a blue emission wavelength of about 440 nm The blue PL emission of the NPCQD was quenched by the Hg^2+^ ion, as provided in Appendix’s Fig. S1. The quantum yield of the NPCQDs was obtained against quinine sulphate as the standard fluorescent material to be equal to 59%. The relatively high QY of the NPCQDs imply that they are well surface-passivated, by carboxyl and hydroxyl groups on the edges and polymeric molecules on the surface of the quantum dots, and as the result of the nitrogen and phosphorus doping, which can effectively modulate the chemical and electrical structure of the surface of NPCQDs^22^.

**Figure 1:**
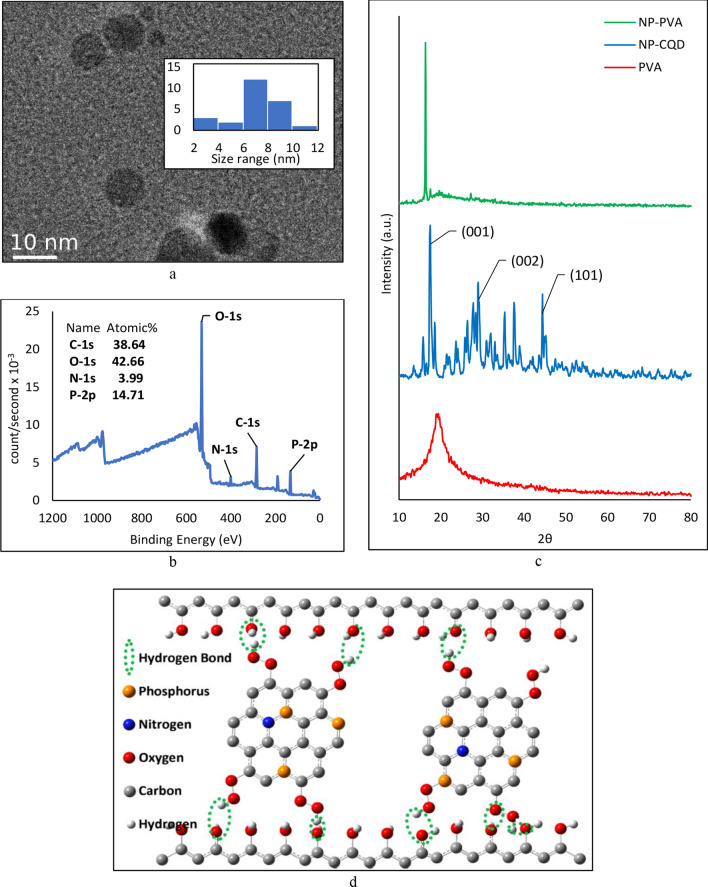
(**a**) TEM of NPCQDs (inset: histogram of size distribution); (**b**) The full XPS diagrams of NPCQDs; (**c**) XRD patterns of PVA, NPCQDs and NP-PVA nanocomposite, and (**d**) Schematic illustration of hydrogen bonding between NPCQDs and PVA in the NP-PVA nanocomposite.

The study of the average particle size and the zeta potential of the NPCQDs was performed in three replications using DLS and high-resolution TEM (Fig. 1-a). The results reveal that the CQDs have an average diameter of 7.67 ± 0.94 nm, with a zeta potential of about − 24.9 ± 1.5 eV. The obtained size distribution is confirmed by the AFM results of the NP-PVA composite. The elemental analysis of the NPCQDs, as depicted by XPS, shown in Fig. 1-b and Fig. S2 (Appendix), reports a higher phosphorous content of 14.7%, compared to 4.0% for nitrogen. Oxygen content is also very high in this NPCQD (42.7%), observed as C=O, P–O and O–C=O bonds, as confirmed by FTIR analysis of the NPCQD (Fig. S2), making it suitable for specific applications.

In pure PVA, as provided in Fig. 1-c, the XRD peak at around 2θ = 19° is attributed to the (101) crystallinity plane, which is rather wide, because of the semi-crystalline nature of PVA. In NPCQDs, aside from the graphitic (001) peak at around 20°, the sp2 (002) and the (101) carbon crystalline peaks are remarkable in the ranges 20°–30°, and 40°–50°, respectively. The crystalline structure of NPCQDs proves a good extent of carbonization in the synthesis process^23^. However, some amounts of polymeric molecules are detectable in the XRD pattern of the NPCQDs, which highlights the polymerization process that occurs as a part of the formation of NPCQDs. In fact, during the synthesis procedure of CQDs, some polymer-like clusters are generally formed as intermediate products, especially on their surface^23^, which also serve as surface passivator to enhance the PL emission of the CQDs^22^.

The XRD pattern of the NP-PVA composite shows an increase at 2θ = 19°. The enhancement of crystallinity is ascribed to the fact that the high number of hydroxyl (O–H) groups in PVA leads to intra- and inter-molecular hydrogen bonding and reordering of NPCQDs and the PVA polymer chains in NP-PVA nanocomposite, thus boosting the crystallinity (Fig. 1-d). The slight shift toward the lower angles indicates that the addition of the NPCQDs to the PVA matrix leads to an increase in the d-spacing value of the lattice constant, showing well dispersity of NPCQDs in the PVA matrix according to Bragg’s equation. The elimination of NPCQDs peaks demonstrates their dissolution in the polymer matrix^23–26^.

### Device evaluation

**Figure 2:**
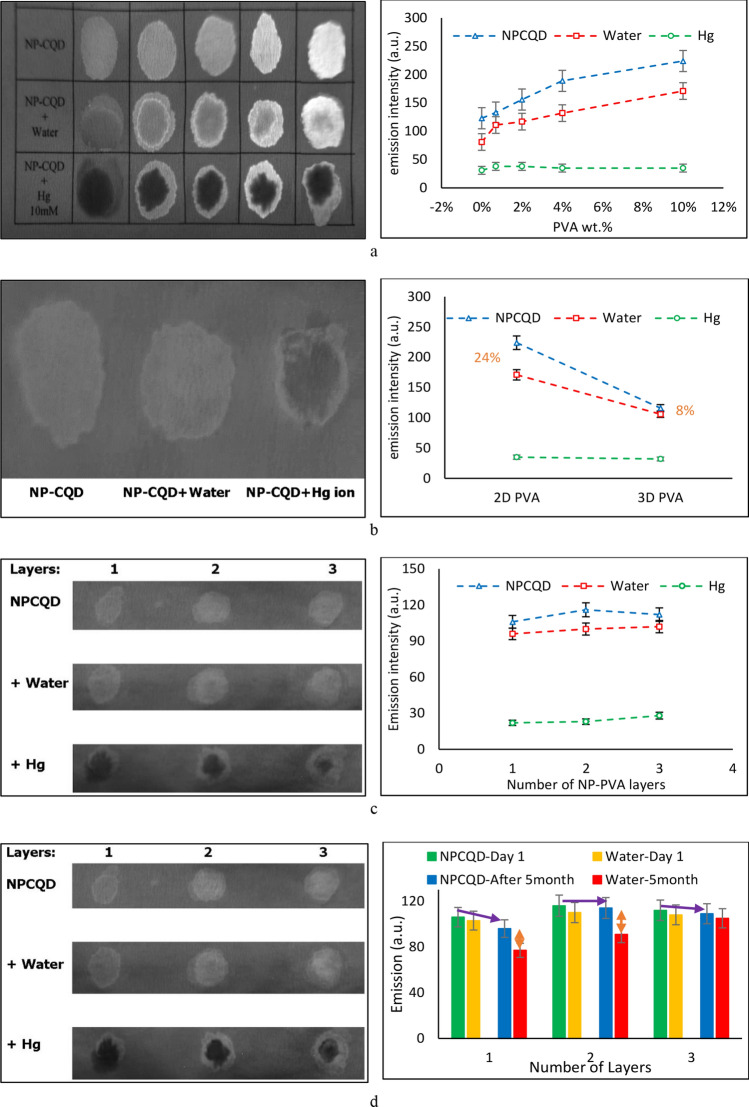
(**a**) The effect of PVA concentration on the emission intensity of the NP-PVA fluorescent spots, and the influence on their emission, when exposed to water and Hg^2+^ drops; (**b**) the controlled FFI after alkaline treatment; and the effect of the pre-alkaline treatment number of NP-PVA layers on the performance of the device (**c**) initially and (**d**) after 5 months.

As described earlier, for the NP-PVA composite synthesis, PVA solutions of different concentrations were prepared and mixed with NPCQDs with an identical volume ratio of 3:1, as 3 (PVA) to 1 (NPCQD) unit volume. As shown in Fig. 2-a, very much stronger emission intensities were achieved as thicker PVA was used as the supporting matrix for the NPCQDs. The reason behind this boosted emission should be sought in more homogenous dispersity of the fluorescent NPCQDs inside the PVA matrix, minimizing the reabsorption effect^27^. As confirmed by XRD results, as a result of the formation of the NP-PVA composite, a significant enhancement was observed in the crystallinity, due to higher numbers of hydrogen bondings between hydroxyl groups of PVA (-OH) and carboxyl (–COOH) groups on the edges of the NPCQDs^28^, leading to rearrangement and reordering of NPCQDs inside PVA chains and thus boosting the crystallinity, and the homogeneity in the dispersity of the NPCQDs. The PVA used in this study, with above 98% of hydrolysis degree, had a large number of hydroxyl groups, making it extensively capable of hydrogen bonding.

However, since the emission of the fluorescent spots was influenced by adding drops of water, it was necessary to think of a method to stop the NPCQDs from flowing with the stream of analyte fluid, as it is poured on the fluorescent spots! This phenomenon, which was termed here as FFI, is necessary to be controlled, especially when dealing with very low concentrations of analyte solution. For that, as described earlier, alkaline treatment of NP-PVA on filter paper was performed to expand PVA and convert it from a 2D fluorescent spot to a 3D PVA matrix that holds the NPCQDs in it, preventing them from moving. Figure 2-b shows how alkaline treatment halts FFI, compared to the right-most column in Fig. 2-a, from 24% PL drop in untreated fluorescent spots to only 8% in alkaline-treated ones. Although the alkaline treatment and the washing steps led to a major reduction in the emission intensity, compared to the initial emission of the fluorescent spots, the change in PL emission as a result of the FFI and addition of Hg^2+^ ion is enough to make the device reliable. The observed change is due to the fact that in physical polymer gels like PVA, physical bindings like the hydrogen bonding here, or Van der Waals interactions in some other polymers, are responsible for the junction point formations. Therefore, with proper modifications, such as the alkaline treatment employed here, the use of these polymers can provide a large extent of volume to facilitate the penetration of the solvent in the structure of the composite^29^. Finally, in order to test the probability of resolving the problem of reduced initial emission, more layers of NP-PVA composites were placed on the paper to make the initial 2D composite thicker. As shown in Fig. 2-c, the alkaline treatment and washing steps reduce the initial emission of the fluorescent spots almost equally, regardless of the initial thickness of the fluorescent spots. However, after leaving the fluorescent PAD in the cold room for several months, a considerable change is observed in the emission strength of the thicker fluorescent spots, showing that it would be a wiser idea to consider thicker initial layers of the fluorescent material when making the device (Fig. 2-d).

#### Hg titration

**Figure 3:**
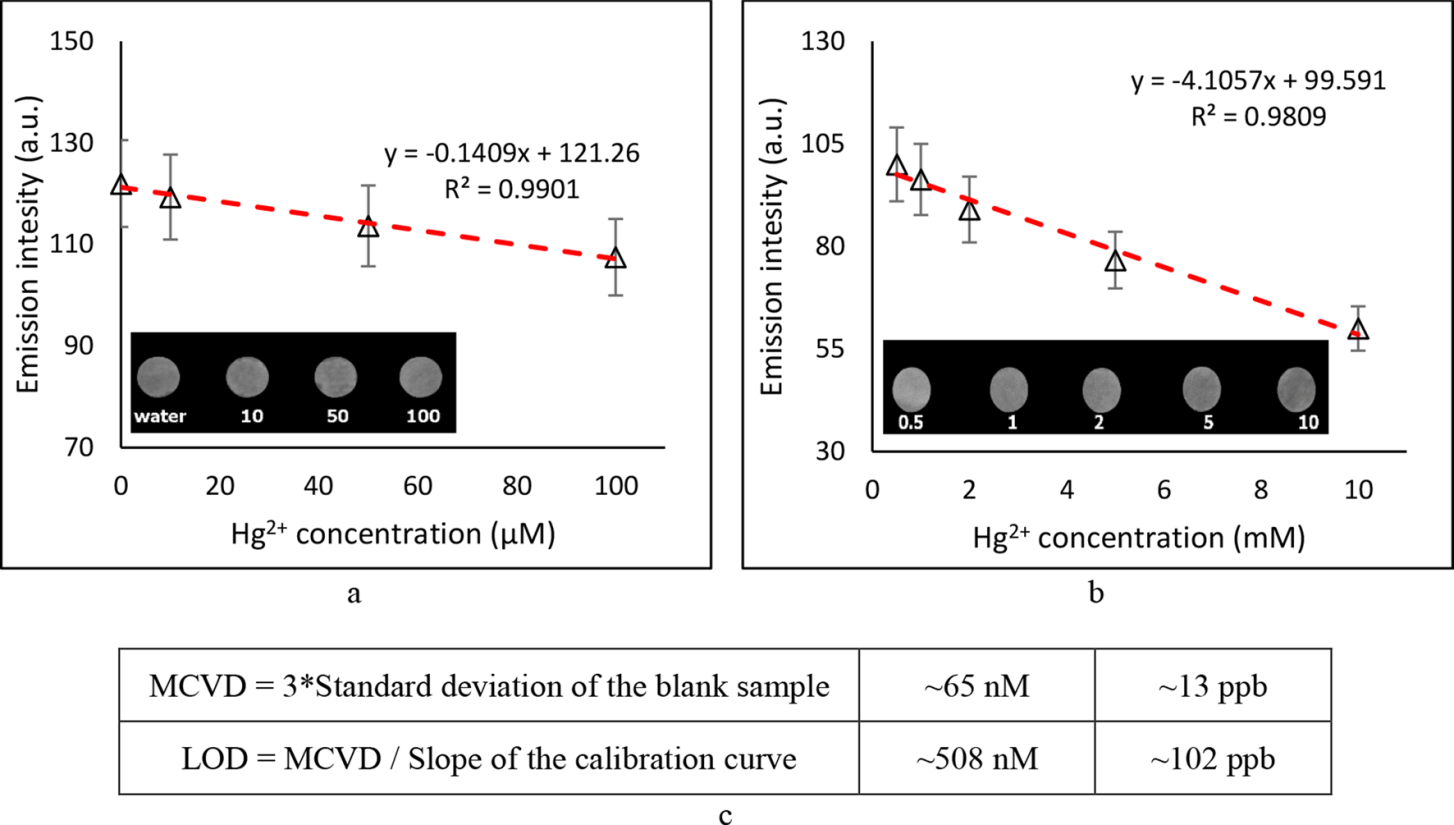
The effect of Hg^2+^ ion titration at two different range orders of (**a**) μM, and (**b**) mM; and (**c**) the minimum concentration of visible detection and LOD of the designed fluorescent PAD.

In order to test the performance of the designed fluorescent PAD, the Hg^2+^ ion was poured at different concentrations on the fluorescent spots. Figure 3 shows the results of the titration at two different linear range orders, namely μM (Fig. 3-a) and mM (Fig. 3-b). According to the obtained results, as shown in Fig. 3-c, the limit of detection (LOD) and the minimum concentration of visible detection (MCVD) of the designed fluorescent PAD was calculated and reported to be about 100 and 13 ppb, respectively.

Previously, there have been limited efforts to make CQD-based fluorescent PADs for the detection of Hg^2+^ ions. However, the reported linear ranges of detection in these investigations were generally very much limited. The linear range of detection is defined as the span of analyte concentration in which signal intensities are linearly proportional to the amount of analyte, which is a very important characteristic of a sensor. However, in 2022, Zou et al. used N-doped CQD-based fluorescent PADs for detecting traces of Hg^2+^ over a reasonably long linear range, i.e. 0–50 μM, which is still half of the achieved linear range of this study, with a LOD of 800 nM. Table 1 shows the reported linear range and the detection limit of several CQD-based PADs. According to the provided data, the technique that was developed in this study led to making a significantly reliable disposable sensor, which can be used by a simple drop-and-detect method.

**Table 1:** Comparison of the LODs of different CQD-based PADs for detection of Hg^2+^.

Method	Linear range (μM)	Detection limit (nM)
l-Ascorbic acid CQD fluorescent PADs^30^	0.00–0.01	2.30
CQD Sponge fluorescent PADs^31^	0–20	26
N-doped CQD fluorescent PADs^32^	0–8	100
N-doped CQD fluorescent PADs^19^	0–50	800
This study: Alkaline-treated NPCQD fluorescent PADs	0–100	508

#### The durability and sustainability

**Figure 4:**
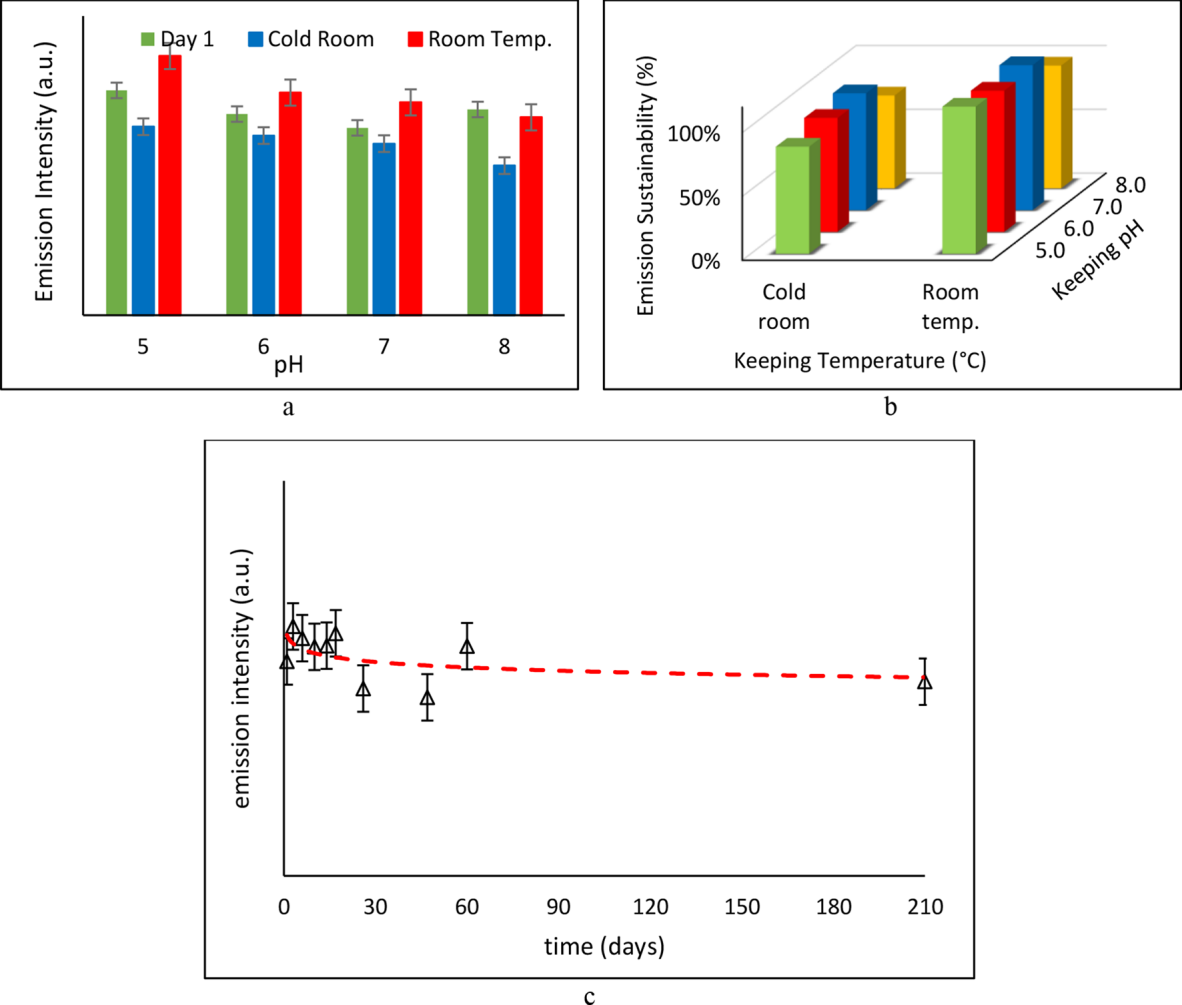
The effect of keeping temperature and pH on (**a**) the durability, and (**b**) the sustainability of the emission intensity after 2 months; (**c**) the emission of the fluorescent PAD’ emission after 7 months.

The durability of the emission intensity of fluorescent spots is defined as how good their initial emission is, and how well it lasts over a several-month period. As shown in Fig. 4-a, the NPCQDs fluorescent spots showed a rather steady and strong emission over several months. However, a remarkable redshift was observed in emission wavelength in NPCQDs kept at room temperature, from blue to green. The apparent increase in the emission intensity records was due to the incapability of the employed software to distinguish emission colours. Hence, based on the results, neutral pH and cold-room conditions were selected as the most proper keeping condition for the designed fluorescent PADs.

Compared to durability, sustainability takes into consideration the impacts of the environmental keeping conditions on how intact the emission intensities of the fluorescent spots remained over several months, regardless of how high it was on the first day of the study. Figure 4-b shows the effect of keeping temperature and pH on the sustainability of the emission. As admitted by the results, about 92% of the emission strength of the NPCQDs fluorescent spots was sustained at neutral pH and cold-room conditions after 2 months. Hence, considering the durability and the sustainability of the emission simultaneously, neutral pH and cold-room conditions are considered the best keeping, leading to reliable performance after several months. Using the data, based on the introduced optimum keeping conditions, the PL emission intensity of the fluorescent spots was recorded after 7 months. Figure 4-c shows the performance of the fluorescent PAD. As it can be noticed, although an emission intensity drop was observed, in the initial days, the designed cost-effective fluorescent PAD is proved to be a reliable device that can be employed for various applications over longer periods.

## Conclusion

Paper-based analytical devices are promising choices for rapid tests and lab-on-chip detection techniques and together with CQDs, they can serve as industrially favourable choices, due to their expanded range of applications, using a simple drop-and-detect method and their cost-effective gram-scale synthesis process. However, there have been limitations in the entrapment of CQDs on cellulose papers in a way that they are not flowed by the stream of analyte fluid, as it is poured on the fluorescent spots, influencing their local PL. Hence, in this investigation, a polyvinyl alcohol/alkaline-based treatment method is systematically generated and developed to minimize the phenomena, termed here as the fluid flow influence. Using this technique, NPCQDs are entrapped inside a 3D crystalline matrix on cellulose paper, in a way the FFI is completely controlled. Despite the major reduction in the emission intensity of the fluorescent spots, compared to their emission before alkaline treatment, the change in PL emission as a result of the FFI and addition of Hg^2+^ ion is enough to make the device reliable and the designed device showed a minimum concentration of visible detection of 13 ppb for the blank sample on which water was poured. The limit of detection of the fluorescent paper-based analytical device for identifying traces of Hg^2+^ was obtained to be about 100 ppb.

Furthermore, due to the stable PL properties of the NPCQDs, the as-designed fluorescent paper-based analytical devices are expected to be functional over several months, showing that about 92% of the emission strength of the NPCQDs fluorescent spots was sustained at neutral pH and cold-room conditions. According to the obtained results, the reliability of the proposed setup was proved for further industrial applications, as cost-effective, fast and environmental-friendly paper-based portable sensors, which can be used directly for a reliable drop-and-detect method, over several months.

## Supplementary Information

Supplementary Information.

## Data Availability

The datasets generated during and/or analysed during the current study are available from the corresponding author upon reasonable request.
