# Supplementary material for: A novel technique to overcome fluid flow influence in carbon quantum dots/paper-based analytical devices
Source: Sci Rep. 2022 Oct 25;12:17861. doi: 10.1038/s41598-022-22837-2 (PMC9596499; doi:10.1038/s41598-022-22837-2)
Supplement: Supplementary file 1 — Supplementary Information. [file 41598_2022_22837_MOESM1_ESM.pdf]

# - Supplementary material -

A Novel Technique to Overcome Fluid Flow Influence in Carbon Quantum Dots/Paper-Based Analytical Devices

Sepideh Zoghi, Moones Rahmandoust*

Protein Research Center, Shahid Beheshti University, Tehran, Iran

* Rahmandoust.moones@gmail.com

Material Characterization

As presented in Figure S1-a, the obtained UV-visible spectrum of the synthesized NPCQDs shows that two maximum absorption wavelengths (dotted red line) at about 235 nm and 345 nm, belonging to the π-π* transition of electrons in aromatic moieties and n-π* of C=O transition, respectively [1,2]. Under an excitation wavelength of 360 nm, NPCQDs, emit a blue emission wavelength of about 440 nm (solid blue line). The NPCQD solution was exposed to seven various 2mM metal cations, namely, Pb^{2+}, Hg^{2+}, Cu^{2+}, Fe^{3+}, Fe^{2+}, Ca^{2+}, and Zn^{2+}. the blue PL emission was better quenched by the Hg^{2+} ion compared to other metal ions, as shown in the inset of Figure S1-a, due to the formation of the mercury hydroxide complex on the surface of NPCQDs [2].

The study of the average particle size and the zeta potential of the NPCQDs was performed in three replications using DLS and TEM. The results reveal that the CQDs are below 10 nm in size, with an average diameter of 7.67±0.94 nm, with a zeta potential of about -24.9±1.5 eV. The obtained size distribution is confirmed by the AFM results of the NPCQDs embedded inside the PVA matrix (NP-PVA), represented in Figure S1-b.

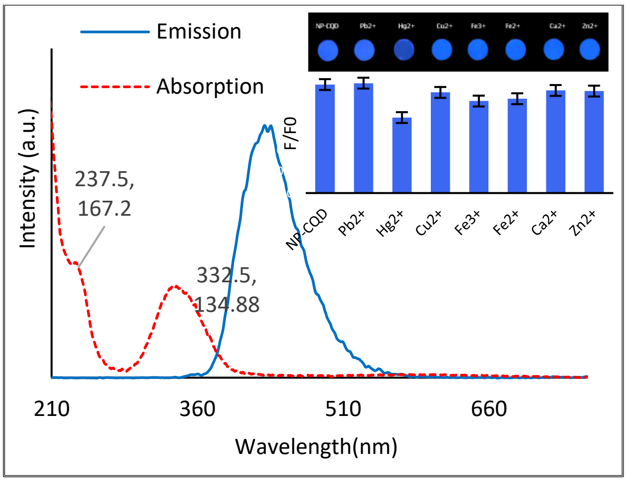

a

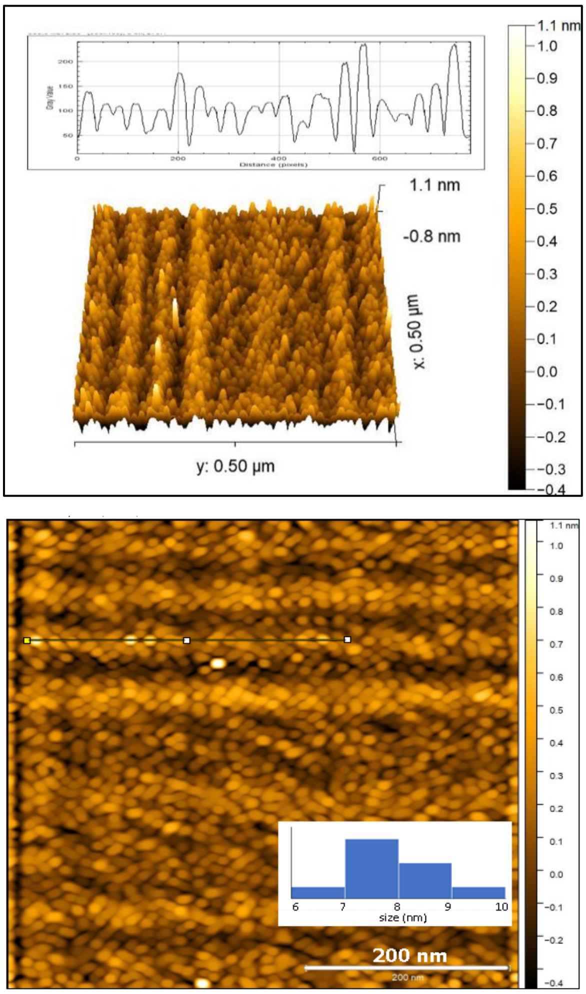

b

Figure S1 (a) The UV-visible spectrum and (inset) the selectivity diagram of the NPCQD; (b) AFM diagram of NPCQDs embedded inside the PVA matrix.

The elemental analysis consists of qualitative and quantitative information about the functional groups of the NPCQDs, as depicted by XPS. As shown in Figures S2-a through S2-e, a higher phosphorous content is observed in the nanoparticle, compared to nitrogen heteroatoms, with 14.7% and 4.0%, respectively. Oxygen content is also very high in this CQD, making it suitable for specific applications. In NPCQDs, the 38.6% carbon content is majorly observed in the form of aromatic C-C sp^2. However, 3D sp^3 C-C bonds are also present. The high oxygen content of 42.7%, is observed in form of C=O, P-O and O-C=O in C-1s, O-1 and P-2p binding energy ranges, as confirmed by FTIR analysis of the CQD. The high-resolution spectra of N1s reveal the presence of two peaks of C-N type at 398.61 eV and 400.20 eV, which could be majorly attributed to pyridinic C-N and lower content of pyrrolic C-N bonds, respectively.

Based on the general fact that the mid-IR spectrum of FTIR (400-4000 cm^{-1}) is divided into four major regions of (1) the fingerprint region (600-1500 cm^{-1}), (2) the double bond region (1500-2000 cm^{-1}), (3) the triple bond region (2000-2500 cm^{-1}), and finally (4) the single bond region, the IR infrared spectrum of the absorption of the NPCQDs, PVA, and NP-PVA samples were studied, as presented in Figure S2-e. In PVA, the symmetric and asymmetric stretching vibrations of CH_2 in the range of 2800-3000 cm^{-1} imply that the polymer hydrocarbon chains take a trans-zigzag conformation and hence the second strong peak at 2922 cm^{-1} arises from the C-H stretching [3]. The presence of a relatively sharp peak at 1700-1800 cm^{-1} indicates the C=O group from the residual acetate, which exists due to the incomplete hydrolysis in PVA. In NPCQDs, on the other hand, the peak at 3570-3200 cm^{-1} / 3460-3100 cm^{-1} belongs to the OH stretching / P-NH_2, whereas, and the P-N / P-O bonds can be distinguished at 1110-930 cm^{-1} / 1080-920 cm^{-1} wavenumbers. The C=C and C-N stretching bonds are observed in the range of 1615-1580 cm^{-1} and 1090-1020 cm^{-1}.

However, the addition of NPCQD to the PVA matrix leads to observing some changes in the intensity or shifts in the position of the peaks. For instance, an increase in the intensity of the absorption at 3276 cm^{-1}, with a slight shift to the right, indicates the presence of OH groups, as it is majorly observed in the PVA and NPCQD, as well as the P-NH_2 stretch in NPCQD. Generally, all changes in the position of the main peaks indicate sufficient interaction between PVA and the NPCQD nanoparticles [4].

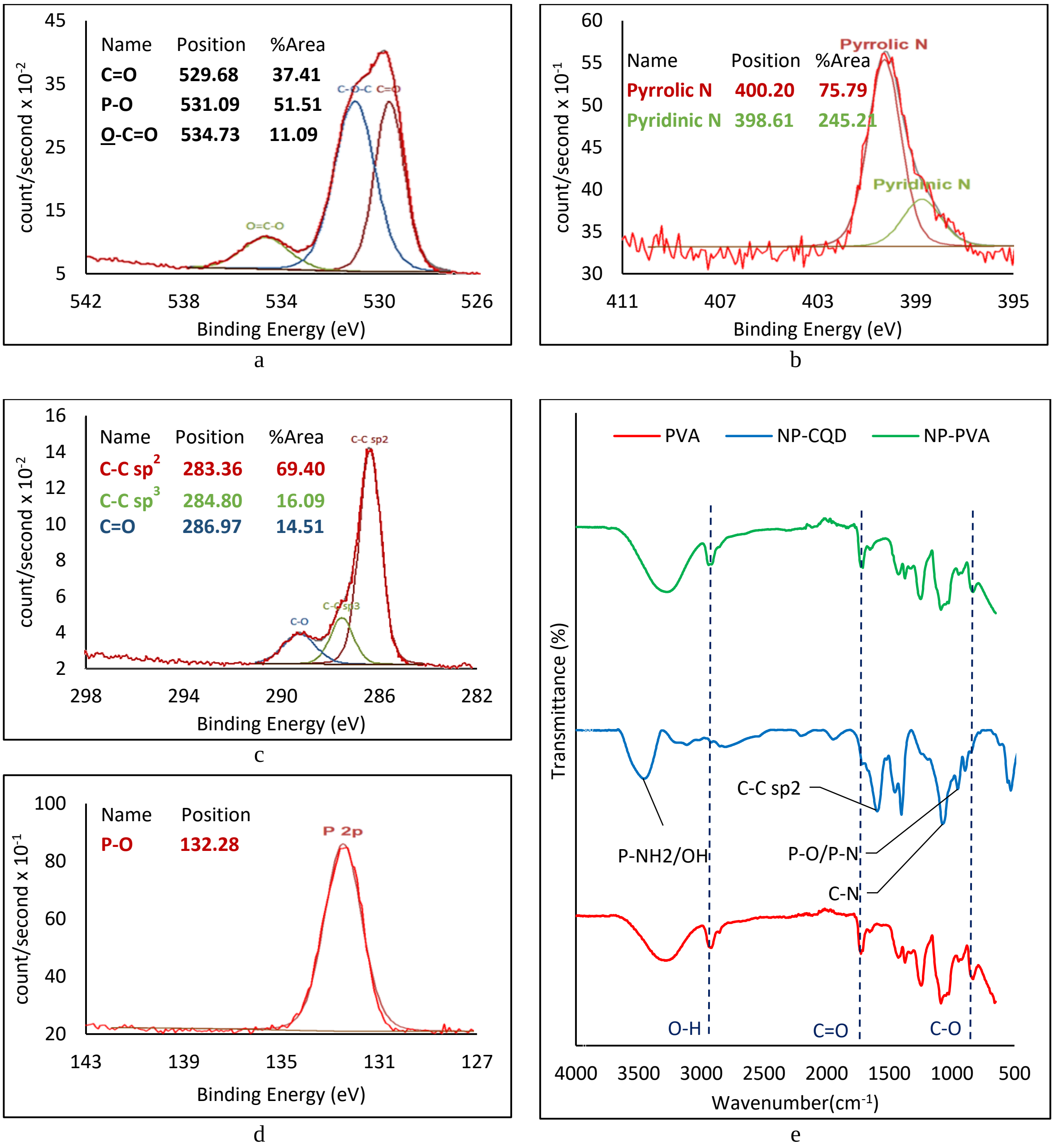

Figure S2 (a) O-1s; (b) N-1s; (c) C-1s; (d) P-2p; and (e) the FTIR spectrum of PVA, NPCQD and NP-PVA nanocomposite.

References

1. Bagheri, Z. *et al.* New insight into the concept of carbonization degree in synthesis of carbon dots to achieve facile smartphone based sensing platform. *Sci. Rep.* **7**, 1–11 (2017).

2. Chandra, S., Laha, D., Pramanik, A. & Chowdhuri, A. R. Synthesis of highly fluorescent nitrogen and phosphorus doped carbon dots for the detection of Fe 3 + ions in cancer cells. *Biol. Chem. Lumin.* 81–87 (2016). doi:10.1002/bio.2927

3. Rogojanu, A., Rusu, E., Olaru, N., Dobromir, M. & Dorohoi, D. O. Development and characterization of poly(vinyl alcohol) matrix for drug release. *Dig. J. Nanomater. Biostructures* **6**, 809–818 (2011).

4. Aziz, S. B. *et al.* Structural and optical characteristics of PVA:C-dot composites: Tuning the absorption of ultra violet (UV) region. *Nanomaterials* **9**, (2019).
